# Synaptic Behaviors in Ferroelectric-Like Field-Effect Transistors with Ultrathin Amorphous HfO_2_ Film

**DOI:** 10.1186/s11671-022-03655-x

**Published:** 2022-01-24

**Authors:** Yue Peng, Wenwu Xiao, Guoqing Zhang, Genquan Han, Yan Liu, Yue Hao

**Affiliations:** 1 State Key Discipline Laboratory of Wide Band Gap Semiconductor Technology, School of Microelectronics, Xidian University, Xi’an, 710071 People’s Republic of China; 2 Xi’an UniIC Semiconductors Co., Ltd., Xi’an, 710075 People’s Republic of China

**Keywords:** FET, HfO_2_, Oxygen vacancy dipole, Memory, Synapse

## Abstract

We demonstrate a non-volatile field-effect transistor (NVFET) with a 3-nm amorphous HfO_2_ dielectric that can simulate the synaptic functions under the difference and repetition of gate voltage (*V*_G_) pulses. Under 100 ns write/erase (W/E) pulse, a memory window greater than 0.56 V and cycling endurance above 10^6^ are obtained. The storied information as short-term plasticity (STP) in the device has a spiking post-synaptic drain current (*I*_D_) that is a response to the *V*_G_ input pulse and spontaneous decay of *I*_D_. A refractory period after the stimuli is observed, during which the *I*_D_ hardly varies with the *V*_G_ well-emulating the bio-synapse behavior. Short-term memory to long-term memory transition, paired-pulse facilitation, and post-tetanic potentiation are realized by adjusting the *V*_G_ pulse waveform and number. The experimental results indicate that the amorphous HfO_2_ NVFET is a potential candidate for artificial bio-synapse applications.

## Background

The need for high density, high performance, and low power consumption has necessitated the development of novel memory devices. Because of their compact structure, non-destructive read-out operation, and multi-bit storage, non-volatile transistors such as ferroelectric field-effect transistors (FeFETs), floating-gate transistors, and IGZO memristors have attracted much attention for embedded memories, computing-in memory, and neuromorphic synapse applications [1–5]. The stimulus is applied to the gate electrode of the transistors for synaptic operation, and the drain side current is the post-synapse current [6, 7].

Recently, non-volatile field-effect transistors (NVFETs) utilizing amorphous Al_2_O_3_ and ZrO_2_ gate insulators were experimentally realized, which was attributed to the switchable polarization (*P*) induced by the voltage-modulation of the oxygen vacancy (${V}_{\mathrm{O}}^{+}$)-related dipoles [8–11]. The mechanism of voltage-modulation of ${V}_{\mathrm{O}}^{+}$ in ferroelectric tunnel junctions was also demonstrated, which improved the tunneling electroresistance ratio of the device [12]. Compared to the polycrystalline doped-HfO_2_ FeFETs, NVFETs with amorphous dielectrics exhibited significantly lower operation voltage and better linearity for multi-threshold voltage operation [9]. These characteristics make them a promising candidate for low-power neuromorphic devices that closely mimic biological behaviors, which are not to be investigated yet.

In this work, biological synapse behaviors such as short-term plasticity (STP), long-term potentiation (LTP), the transition from short-term memory (STM) to long-term memory (LTM), and spike-timing-dependent plasticity (STDP) are emulated based on the single amorphous HfO_2_ NVFET, without using additional circuit elements.

## Methods

The process flow in [9] was used to fabricate the NVFETs with an amorphous HfO_2_ gate insulator on 4-inch n-type Ge(001). After the pre-gate cleaning, the substrate was loaded into an atomic layer deposition (ALD) chamber to deposit the HfO_2_ at 300 °C. Then, a 100-nm-thick TaN gate electrode was deposited by the reactive sputtering. After the gate electrode patterning and etching, the source/drain (S/D) regions were implanted by BF_2_^+^. 20-nm thick nickel (Ni) S/D metal electrodes were formed by a lift-off process. Finally, the repaid thermal annealing (RTA) at 350 °C was carried out to improve the interface quality and form the Ni germanium silicide S/D contacts.

**Fig. 1:**
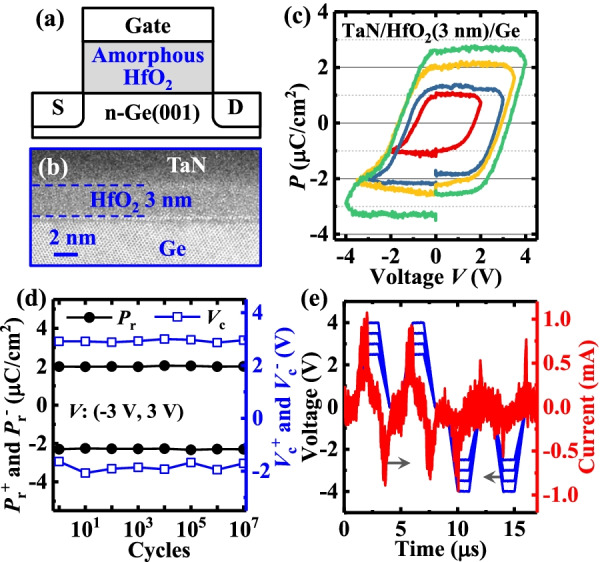
**a** Schematic of the NVFET with amorphous HfO_2_ gate insulator. **b** HRTEM image shows the 3-nm-thick amorphous HfO_2_. **c** Measured *P–V* curves for TaN/HfO_2_/Ge capacitor. **d** *P*_r_ and *V*_c_ *vs.* the number of sweeping cycles for amorphous HfO_2_ capacitors. **e** PUND test of HfO_2_ capacitor exhibiting the switching current component by isolating the non-switching charge

The schematic of the fabricated NVFET is shown in Fig. 1a. Figure 1b shows a 3-nm-thick amorphous HfO_2_ imaged with high-resolution transmission electron microscopy (HRTEM). Figure 1c depicts the measured ferroelectric-like *P vs.* voltage (*V*) behavior in the amorphous HfO_2_ capacitor at a frequency of 1 kHz. The underlying mechanism for the ferroelectric-like behaviors in this amorphous HfO_2_ devices is similar to that for those devices in Refs. [8, 9]. The extracted evolution of the remnant *P* (*P*_r_) and coercive voltage (*V*_c_) for the device during the endurance test is shown in Fig. 1d. No wake-up or imprint is observed over 10^6^ cycles. A positive-up and negative-down (PUND) test is used to extract the switching current component of the device by isolating the non-switching charge (Fig. 1e), demonstrating the true *P*.

## Results and Discussion

**Fig. 2:**
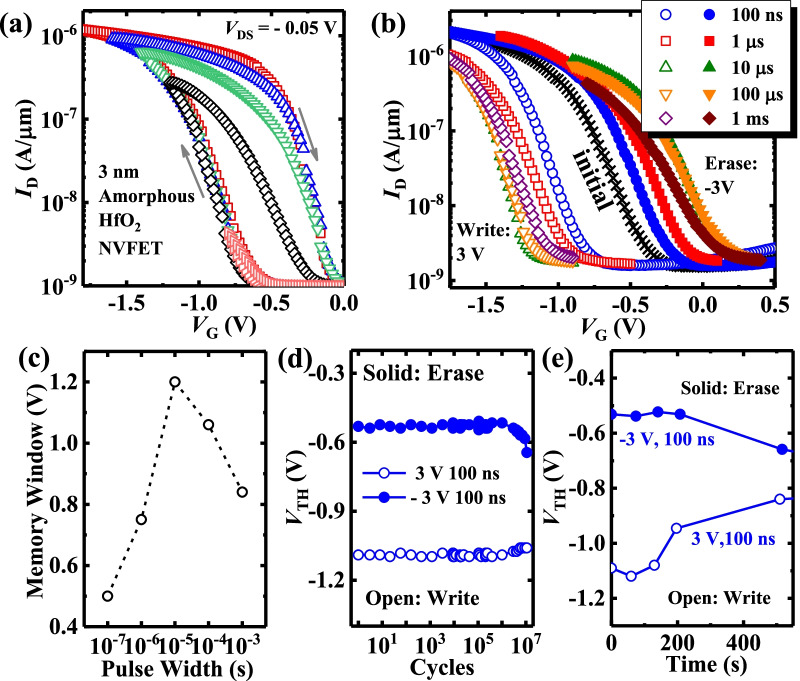
**a** Dual-direction sweeping of *I*_D_-*V*_G_ curves of the amorphous HfO_2_ NVFET. **b** Measured *I*_D_-*V*_G_ curves of the device with ± 3 V W/E pulses, and the pulse width varies from 100 ns to 1 ms. **c** MW for the amorphous HfO_2_ NVFET with various pulse width. **d** Stable MW maintains after 10^6^ W/E cycles underwent ± 3 V, 100 ns W/E pulses. **e** Several hundred seconds retention time was maintained of the amorphous HfO_2_ device

In contrast to the trapping/detrapping process [13–15], a ferroelectric-like clockwise hysteresis loop is observed for the DC sweeping of the drain current (*I*_D_) as a function of gate voltage (*V*_G_) curves for the transistor with the amorphous HfO_2_ gate insulator, as shown in Fig. 2a. The non-volatile memory function is induced by the ferroelectric-like *P* switching in the gate stack. Figure 2b shows the initial *I*_D_–*V*_G_ curve for the device and those underwent with 100 ns, 1 μs, 10 μs, 100 μs, and 1 ms write/erase (W/E) pules at ± 3 V voltage providing a non-volatile memory function, respectively. The device has a gate length (*L*_G_) of 3 μm and a gate width (*W*) of 80 μm. The write (erase) operation is achieved by applying positive (negative) voltage pulses to the gate of the HfO_2_ FET, to raise (lower) its threshold voltage (*V*_TH_).

Figure 2c plots the MW values for different W/E pulse widths. As the pulse width increases from 100 ns to 10 μs, the MW increases to 1.2 V; but when the W/E pulse width further increases, the MW decreases. Trapping/detrapping process is thought to cause the degradation of MW under the 100 μs to 1 ms W/E pulses. Here, MW is the *V*_TH_ difference between the two states, and *V*_TH_ is defined as *V*_G_ at *I*_D_ = 100 nA⋅*W*/*L*_G_.

As shown in Fig. 2d, a stable MW is maintained over 10^6^ W/E cycles. Figure 2e shows that a stable MW of the amorphous HfO_2_ device can be maintained over several hundred seconds. The limitation retention time of the device is mainly due to the smaller *P*_r_ and large depolarization field. Recent studies have shown that the non-volatile devices with limited retention time can be alternative candidates for high-density and lower power DRAM architectures [16, 17].

**Fig. 3:**
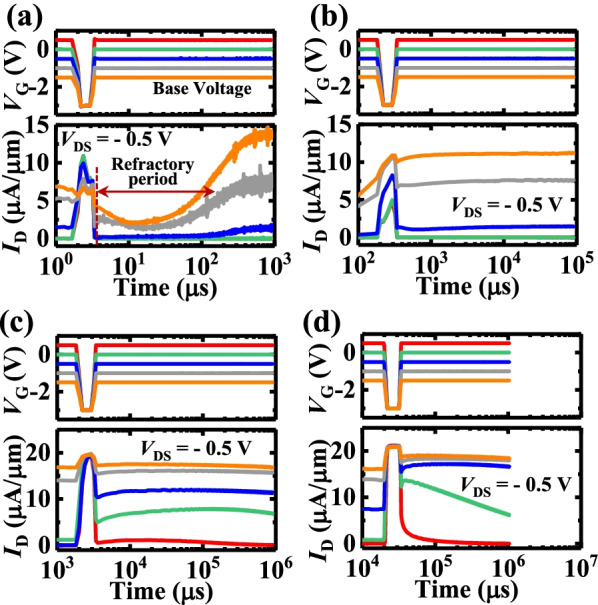
Base voltage varies from 0.5 to -1.5 V. The widths of *V*_G_ pulses in **a**–**d** are 1 μs, 100 μs, 1 ms, and 10 ms, respectively

Synapse is a basic unit of the human neural network to realize the information transmission from the pre-synaptic neuron to the post-synaptic neuron. The STP is a key factor that affects the biographic performance of the NVFET synapse in the neural system [18]. Figure 3 shows the STP characteristics of a HfO_2_ NVFET under the single *V*_G_ pulse with a fixed pulse magnitude of -3 V. The *V*_G_ pulse width varies from 1 μs to 10 ms and the base voltage varies from 0.5 V to − 1.5 V. As a three-terminal device, the STP performance can be modulated by changing the base voltage, magnitude, and width of the *V*_G_ pulses. Underwent an applied *V*_G_ stimulus, the post-synaptic *I*_D_ of the device increases to a high *I*_D_ state and decays to a low *I*_D_ state when the *V*_G_ pulse ended. For all the measurements, the devices are in the same relaxed pre-state.

As shown in Fig. 3a, underwent a 1 μs *V*_G_ pulse, the device exhibits a lower post-synaptic *I*_D_ under the base voltage of − 1.5 V and − 1.0 V compared to the cases under 0 V and − 0.5 V *V*_G_ base. It is speculated that this could be due to the smaller difference between base and pulse *V*_G_ voltages. As the *V*_G_ pulse is widened to ms, the post-synaptic *I*_D_ no longer depends on the base voltage (Fig. 3c, d). In general, the post-synaptic *I*_D_ of the device is improved with widening the stimulus *V*_G_ pulse.

**Fig. 4:**
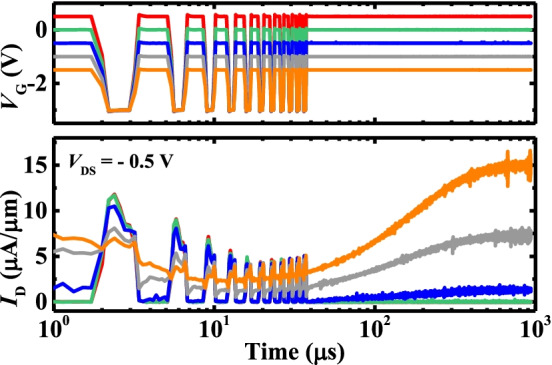
Post-synaptic *I*_D_ of the device underwent multiple *V*_G_ input pulses within the relative refractory period

According to Fig. 3, there is a refractory period after the *V*_G_ pulse. The *I*_D_ barely varies with the *V*_G_, which accurately simulates the bio-synapse with the external stimulating signal. The refractory period of the NVFET synapse is approximately 10–100 μs, which does not depend on the *V*_G_ pulse width or magnitude. Figure 4 depicts the post-synaptic *I*_D_ of the transistor that underwent multiple *V*_G_ input pulses within the refractory period. During this period, *I*_D_ is excitable by the *V*_G_ pulse, but its value is less than that for the initial pulse firing. After the refractory period, the post-synaptic *I*_D_ increases with time to a saturate state, and values of post-synaptic *I*_D_ in saturation increase with the decrease in the base voltage.

**Fig. 5:**
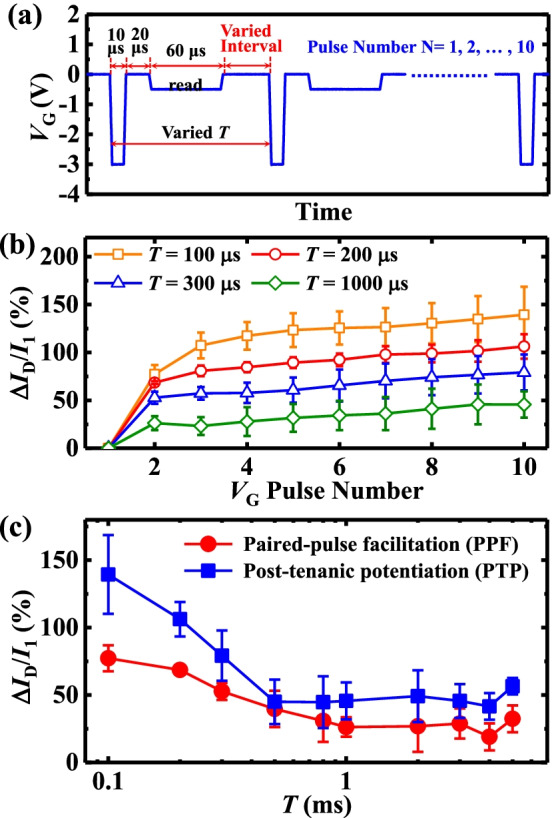
**a** *V*_G_ pulse waveform with the different *T*. **b** The *I*_D_ increase as a function of the gate stimulus number plots with the different *T*. **c** Extracted (*I*_2_ − *I*_1_)/*I*_1_ and (*I*_10_ − *I*_1_)/*I*_1_, representing PPF and PTP behaviors, respectively, of the transistor

Besides the width and magnitude of the pulse, the stimulation rate also influences the memory formation of the device. To examine the effects of the stimulation rate on the transistor, the ten cycles (*N* = 10) of stimuli/read *V*_G_ are applied to the gate electrode. As shown in Fig. 5a, during the stimuli or read, the amplitude and time of the *V*_G_ pulse are fixed, and the cycle period *T* is changed by varying the interval parameter. The *I*_D_ of the transistor was read at low voltage immediately after each stimulation pulse, which is denoted by *I*_1_, *I*_2_, …, *I*_10_ [19].

The dynamic change in the *I*_D_ of the amorphous HfO_2_ NVFET under a series of *V*_G_ pulses with the different *T* at a *V*_DS_ of − 0.5 V is shown in Fig. 5b. The *I*_D_ (i.e., Δ*I*_D_/*I*_1_) of the device increases with the stimuli *T* numbers to mimic the memory behavior in the biological system. Here, Δ*I*_D_ is calculated by *I*_*N*_ − *I*_1_, (*N* = 1, 2, …, 10). Note that the *I*_D_ of the device increases, i.e., (Δ*I*_D_/*I*_1_) with the reduced *T*. With a high stimulation rate being the most effective and a low stimulation rate being the least effective for transforming from STM to LTM.

Figure 5c plots the (*I*_2_ − *I*_1_)/*I*_1_ and (*I*_10_ − *I*_1_)/*I*_1_, which represent the experimental conditions for paired-pulse facilitation (PPF) and post-tetanic potentiation (PTP) used in biological studies, respectively [20, 21]. The PPF and PTP phenomena in our amorphous HfO_2_ synaptic transistor can be compared to synapses in biology. If be former, the synaptic response is enhanced when one stimulus is followed by the same stimulus soon after; if be latter, the synaptic transmission gradually increases with the number of stimuli when a series of stimuli are received [20–22]. These verify the feasibility of the amorphous HfO_2_ device in realizing the transformation of simulated biological memory. The error bars reflect the standard deviation when repeating the measurement a few times to prove the correctness of the data and minimize fluctuations in data.

**Fig. 6:**
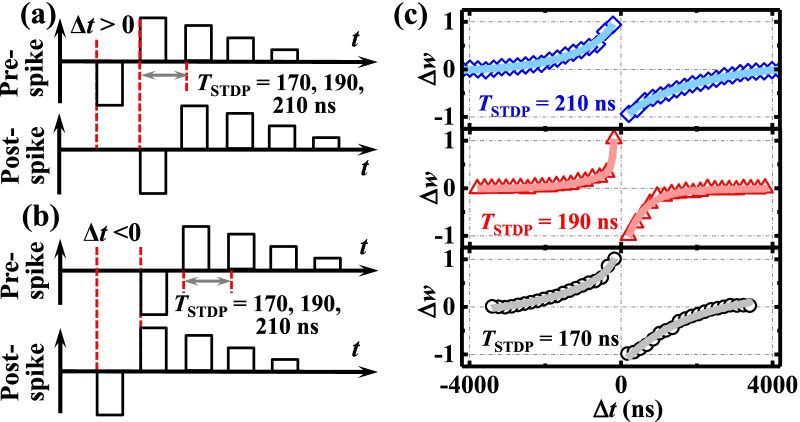
**a** and **b** Spike timing waveform for the implementation of STDP. **c** Measured STDP curves in the amorphous HfO_2_ synaptic transistor with different spike periods (*T* = 170, 190, and 210 ns)

The temporal relationship of activity between the pre- and post-synaptic neurons is another important aspect of the synapse. We define *t*_PRE_ and *t*_POST_ as the arrival times of the pre-spike and the post-spike, respectively. The change in synaptic weight (Δ*w*) is a function of the Δ*t* (Δ*t* = *t*_PRE_ − *t*_POST_) between pre- and post-synaptic activity [23]. For a given stimulation, LTD will occur if Δ*t* > 0, while LTP will occur if Δ*t* < 0. STDP is defined as the change in synaptic weight of the Δ*t* between pre- and post-synaptic activity. By utilizing the waveforms adopted in Fig. 6a, b, the STDP curves for the amorphous HfO_2_ NVFET-based synapse are extracted with 100 ns spikes and shown in Fig. 6c. The pre-and the post-spike resembling the output of the leaky integrate-and-fire neurons are constitutive of an initial negative pulse followed by a sequence of positive pulses with the decreased amplitude. As shown in Fig. 6c, the amorphous HfO_2_ NVFET can stimulate the STDP learning rule successively with spiking period time *T*_STDP_ varying from 170 to 210 ns. The HfO_2_ NVFET obtains a steeper conductivity change around Δ*t* = 0 at the *T*_STDP_ = 190 ns compared to the other *T*_STDP_ conditions, which is possibly due to the better matching between the spike waveform shape applied at the gate electrode and the non-volatile characteristics induced by ferroelectric-like behavior of the device.

## Conclusions

In this work, we report an ultrathin amorphous HfO_2_ NVFET to emulate the bio-synapse. An MW of 0.56 V with an endurance above 10^6^ cycles is experimentally demonstrated under the ± 3 V and 100 ns W/E pulses. Furthermore, various synaptic behaviors including STP under different stimuli, transitioning from STM to LTM, PPF, PTP, and STDP performance are realized in the device.

## Data Availability

The datasets supporting the conclusions of this article are included in the article.

## Abbreviations

TaN: Tantalum nitride
*P*_r_: Remnant polarization
*E*_c_: Coercive electric field
Ge: Germanium
ALD: Atomic layer deposition
BF_2_^+^: Boron fluoride ion
HfO_2_: Hafnium oxide
GeO_*x*_: Germanium oxide
HRTEM: High-resolution transmission electron microscope
Ni: Nickel
RTA: Repaid thermal annealing
MW: Memory window
*I*_D_: Drain current
Δ*w*: Synaptic weight
*V*_G_: Gate voltage
*V*_TH_: Threshold voltage
NVFET: Non-volatile field-effect transistor
T: Period
FeFET: Ferroelectric field-effect transistor
STDP: Spike-timing-dependent plasticity
STP: Short-term plasticity
LTP: Long-term potentiation
STM: Short-term memory
LTM: Long-term memory
PPF: Paired-pulse facilitation
PTP: Post-tetanic potentiation
